# Acquired Diverticulosis of the Entire Colon in a Cadaver

**DOI:** 10.7759/cureus.10511

**Published:** 2020-09-17

**Authors:** Jean-Marc P Lucas, Carey A Roberts, Carly A Gunderson, Francis J Liuzzi, Oren D Rosenthal

**Affiliations:** 1 Anatomy, Lake Erie College of Osteopathic Medicine, Bradenton, USA; 2 Family Medicine, Naval Hospital Jacksonville, Jacksonville, USA; 3 Pediatrics, Nemours Children's Hospital, Orlando, USA

**Keywords:** pancolonic diverticulosis, diverticula, diverticulum, colonic diverticulum, cadaver case report, diverticula number, diverticular disease, large bowel diverticula, diverticula of colon, diverticulitis

## Abstract

Diverticulosis involving the entire colon is rare in Western society. During a routine dissection of a 74-year-old Caucasian female cadaver, who died from vascular disease complications, diverticula were observed in the ascending, transverse, and descending colon. A total of 413 diverticula were manually counted. The majority of diverticula arose from the right and transverse colon, which is atypical of the disease in Western society. Histological examination of sections from sample diverticula reveals morphology consistent with pseudodiverticula, suggestive of acquired disease. Pancolonic diverticulosis may be associated with systemic diseases such as collagen disorders, vascular complications, and increased risk of recurrent diverticulitis. This case is an example of a rare manifestation of diverticular disease that is important for clinicians to recognize when evaluating and treating patients with gastrointestinal symptoms.

## Introduction

Diverticulosis is the presence of intestinal mucosal and submucosal outpouchings that communicate with the lumen of the colon [1]. Diverticula arise as a result of high intracolonic pressure that causes weak points in the muscularis propria to flare outwards [2]. Diverticulosis is primarily a disease of Western culture and mostly occurs in the sigmoid colon [2,3]. Diverticulosis has been reported to occur in 42% of adults living in Western societies, with a left-sided predominance [2]. In Asia and Africa, diverticulosis is relatively rare, and when present, occurs chiefly in the right colon [4,5]. When it occurs in all three regions of the colon it is referred to as pancolonic [6], the prevalence of which is 7% in western culture [7].

Peery et al. report that people with diverticulosis have an average of 14 diverticula, although there appears to be a large degree of variance within populations [8]. The number of diverticula present increases with a patient’s age [8]. Race, however, is not a factor in diverticula number [8]. Dickerson et al. found a positive correlation between diverticula number and severity of diverticulosis [9].

There is limited literature available on pancolonic diverticulosis as well as the number of diverticula associated with individual cases. The purpose of this case study is to address this gap in the literature by quantifying the overall number of diverticula and reporting their distribution throughout the colon in an individual with pancolonic diverticulosis.

## Case presentation

**Figure 1:**
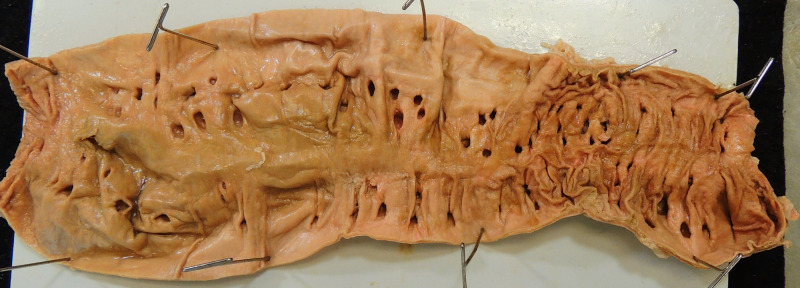
A section of the ascending colon with diffuse diverticula.

**Figure 2:**
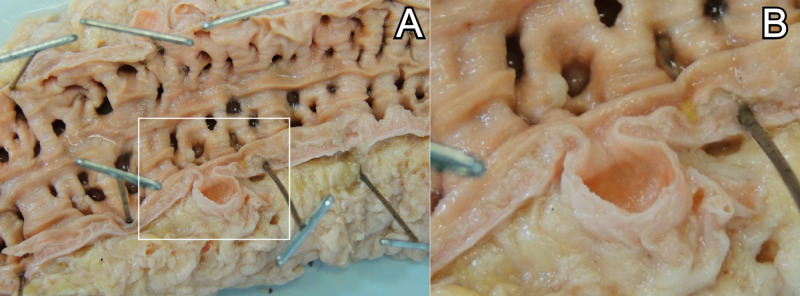
(A) A section of the descending colon with diffuse diverticula. One transected diverticulum is indicated by the box. (B) A magnified image of the transected diverticulum.

A 74-year-old Caucasian female cadaver with a history of peripheral vascular disease, coronary artery disease, and a left above-knee amputation underwent routine dissection. At first, diverticula were observed in the transverse colon. Further dissection revealed diverticula along the length of the colon. The entire colon was dissected free, opened along its long axis, and flushed. Adipose tissue was resected and the colon was cut into seven sections for diverticula counting. Sections 1 and 2 were taken from the ascending (right) colon. Sections 3, 4, and 5 were taken from the transverse colon. Sections 6 and 7 were taken from the descending (left) colon. Sections of the ascending and descending colon are shown in Figure 1 and Figure 2, respectively.

**Figure 3:**
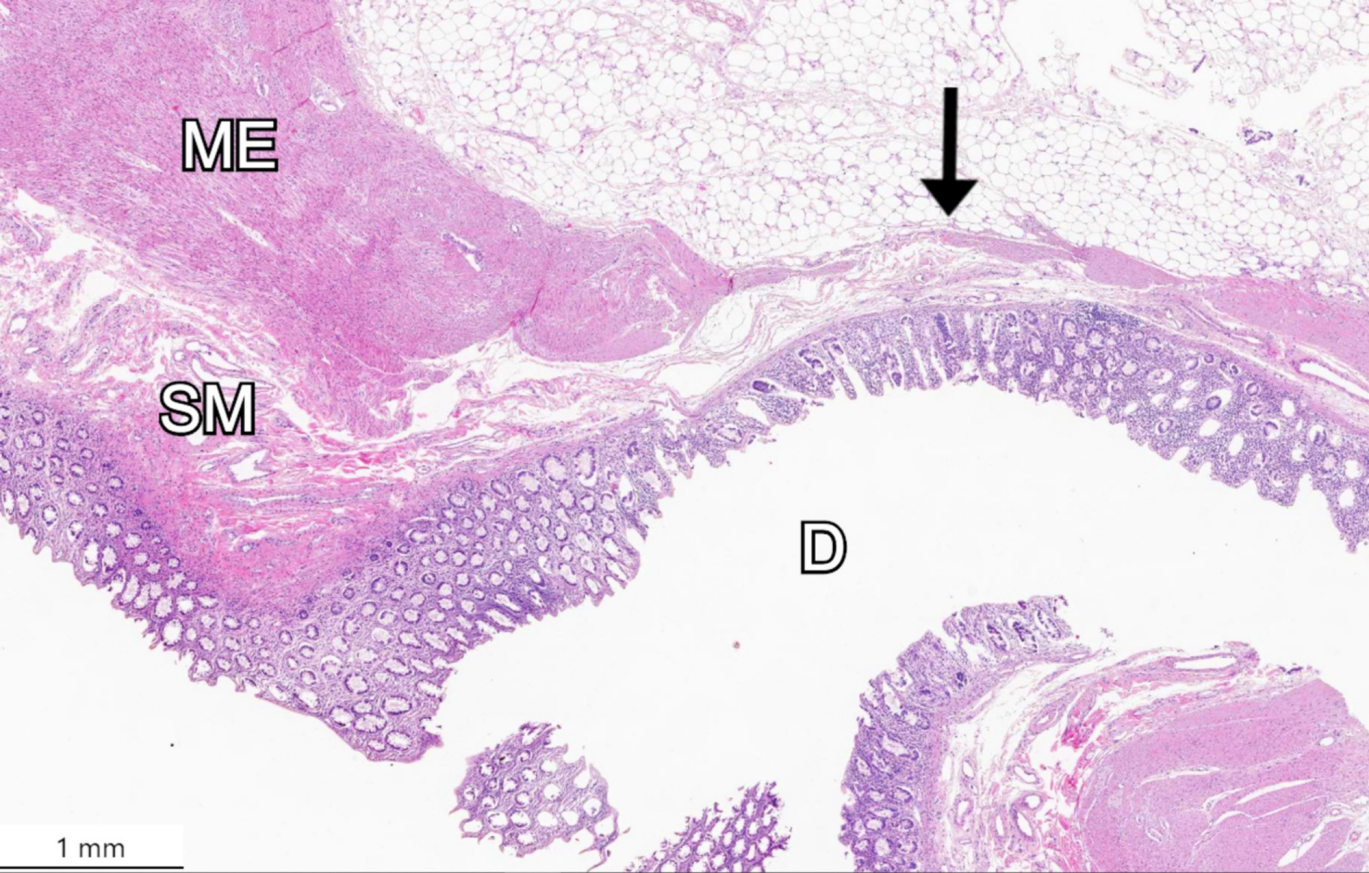
Hematoxylin and eosin (H&E) staining of a diverticulum with thinning submucosa and muscularis externa, as denoted by the arrow. D: diverticulum. ME: muscularis externa. SM: submucosa.

Colonic sections were opened to optimally display the lumen for accurate counting. Plastic markers were used to track the diverticula during the manual count. Additionally, samples of diverticula were prepared and processed for hematoxylin and eosin (H&E) staining (Figure 3).

Microscopic examination revealed a marked thinning of the muscularis externa in the region of the diverticula, which is consistent with acquired diverticulosis (also known as pseudodiverticula). It further revealed attenuated muscularis externa surrounding diverticula with normal colonic mucosa. The authors counted 413 diverticula throughout the colon. The ascending, transverse, and descending colon sections had 168, 195, and 50 diverticula, respectively. The individual section with the highest density of diverticula was section 5 (distal transverse colon). The section with the lowest density of diverticula was section 7 (sigmoid colon). Diverticula number from each section are reported in Table 1.

**Table 1:** Total number of diverticula in each colonic section and segment.

Section	Section Count	Colon Segments	Segment Count
1	86	Right (Ascending)	168
2	82		
3	44	Transverse	195
4	60		
5	91		
6	41	Left (Descending)	50
7	9		

## Discussion

This individual’s diverticula were mostly localized to the ascending and transverse colon, which is consistent with diverticulosis in Asian and African populations [4,5]. Diverticular disease has an estimated genetic contribution of 40% to 53% [10]. The presence of extensive diverticula in this individual could be linked to a genetic predisposition for diverticulosis exacerbated by additional lifestyle risk factors [10]. Specifically, collagen cross-linking defects are speculated to play a role in diverticulosis cases with high diverticula quantity [2,8]. Ehlers-Danlos (type IV), Marfan, Coffin-Lowry, and Williams syndromes are associated with both vascular disease and relatively severe diverticulosis [2,10,11]. Increased expression of matrix metalloproteinase-1 has been implicated in cases of diverticulosis without diverticulitis [10]. Patients with pancolonic diverticula may warrant genetic evaluation to rule-out collagen disorders.

Presenting symptoms may be different in patients depending on the number of diverticula, as well as the section of colon in which the diverticula are present [9]. Diverticulosis is usually asymptomatic, but may present with hematochezia, abdominal pain localized to the affected colonic segment, alterations in bowel movements, and diverticulitis [2]. These symptoms may resemble other abdominal pathology, such as inflammatory bowel disease (IBD) [12]. The prevalence and severity of these symptoms may be exacerbated in cases of pancolonic diverticulosis. As such, abdominal pain in pancolonic diverticulosis may be diffuse instead of localized to a single abdominal quadrant, which may appear as irritable bowel syndrome (IBS) on initial presentation [13]. Clinicians should consider pancolonic diverticulosis in patients presenting with diffuse abdominal pain, particularly when complicated with hematochezia and family history of diverticular disease.

Diverticular disease may induce chronic systemic inflammation, which can lead to vascular disease culminating in myocardial infarction and stroke [10,14]. This individual’s cause of death was from complications of peripheral vascular disease. One could speculate this individual’s vascular morbidity and mortality may be linked to diffuse colonic diverticular disease in association with systemic inflammation [10,14]. Patients with pancolonic diverticula should be closely monitored for the development of vascular disease.

Treatment options differ depending on the extent of diverticular disease and symptomatic presentation. Treatment ranges from observation for diverticula-associated abdominal pain to colectomy for recurrent diverticulitis [15]. Al Harakeh et al. found that pancolonic diverticulosis is associated with a medium risk of recurrent diverticulitis [16]. This finding suggests that the pathogenesis of recurrent diverticulitis is multifactorial and not solely dependent on diverticula quantity. Though pancolonic diverticulosis does not guarantee the progression to recurrent diverticulitis, clinicians should be wary of the possibility when dealing with diverticula to this magnitude.

## Conclusions

Few studies have reported diverticulosis throughout the entire colon. Even fewer studies have reported the number of diverticula associated with individual diverticulosis cases. This case report demonstrates a rare case of pancolonic diverticulosis with 413 diverticula. Pancolonic diverticulosis may be associated with increased overall disease severity, collagen disorders, vascular complications, and increased risk for developing recurrent diverticulitis. As such, the findings presented in this case are relevant to clinicians screening and treating patients for pancolonic diverticulosis.
